# Putative Epigenetic Involvement of the Endocannabinoid System in Anxiety- and Depression-Related Behaviors Caused by Nicotine as a Stressor

**DOI:** 10.1371/journal.pone.0158950

**Published:** 2016-07-12

**Authors:** Tamaki Hayase

**Affiliations:** Department of Legal Medicine, Kyoto University, Kyoto 606–8501, Japan; Peking University, CHINA

## Abstract

Like various stressors, the addictive use of nicotine (NC) is associated with emotional symptoms such as anxiety and depression, although the underlying mechanisms have not yet been fully elucidated due to the complicated involvement of target neurotransmitter systems. In the elicitation of these emotional symptoms, the fundamental involvement of epigenetic mechanisms such as histone acetylation has recently been suggested. Furthermore, among the interacting neurotransmitter systems implicated in the effects of NC and stressors, the endocannabinoid (ECB) system is considered to contribute indispensably to anxiety and depression. In the present study, the epigenetic involvement of histone acetylation induced by histone deacetylase (HDAC) inhibitors was investigated in anxiety- and depression-related behavioral alterations caused by NC and/or immobilization stress (IM). Moreover, based on the contributing roles of the ECB system, the interacting influence of ECB ligands on the effects of HDAC inhibitors was evaluated in order to examine epigenetic therapeutic interventions. Anxiety-like (elevated plus-maze test) and depression-like (forced swimming test) behaviors, which were observed in mice treated with repeated (4 days) NC (subcutaneous 0.8 mg/kg) and/or IM (10 min), were blocked by the HDAC inhibitors sodium butyrate (SB) and valproic acid (VA). The cannabinoid type 1 (CB1) agonist ACPA (arachidonylcyclopropylamide; AC) also antagonized these behaviors. Conversely, the CB1 antagonist SR 141716A (SR), which counteracted the effects of AC, attenuated the anxiolytic-like effects of the HDAC inhibitors commonly in the NC and/or IM groups. SR also attenuated the antidepressant-like effects of the HDAC inhibitors, most notably in the IM group. From these results, the combined involvement of histone acetylation and ECB system was shown in anxiety- and depression-related behaviors. In the NC treatment groups, the limited influence of SR against the HDAC inhibitor-induced antidepressant-like effects may reflect the characteristic involvement of histone acetylation within the NC-related neurotransmitter systems other than the ECB system.

## Introduction

Tobacco use has been the leading global cause of preventable death due to a number of chronic diseases (e.g. cancer and lung/cardiovascular diseases), and is associated with lethality in approximately 6 million people every year [1, 2]. The addictive use of tobacco is sustained due to nicotine (NC), a highly addictive psychoactive ingredient [1], and the chronic use of NC has been reported to result in increased emotional symptoms such as anxiety and depression [3, 4]. Anxiety and depression are representatively observed as withdrawal symptoms in dependent smokers [5–7]. Furthermore, in some daily smokers, direct anxiogenic and depressogenic effects, which disappear following smoking cessation, have been reported [8–10], and the involvement of the combined activation and desensitization of nicotinic acetylcholine receptors (nAChRs) was suggested in the direct causal link between smoking and emotional symptoms using several rodent experimental models [11, 12]. On the other hand, NC-induced anxiolytic and antidepressant effects have also been reported depending on the experimental model, the route of NC administration and the time course of administration [3, 13–17], and these effects are thought to characteristically reinforce the habitual use of NC.

Anxiety and depression are also observed as frequent psychiatric outcomes of various stressors in humans and associated with inappropriate regulation of brain stress systems [18, 19]. In addictive smokers, the dysregulated stress response in the brain similar to cases exposed to stressors has been reported and stressor-like effects of NC were demonstrated [3, 4, 20]. Furthermore, in several epidemiological and experimental studies, exacerbation of emotional symptoms such as anxiety and depression has been reported in certain stressor-exposed smokers [21–23]. However, depending on the type of NC and/or stressor treatment, stress-related anxiety and depression were decreased by cigarette smoking [24]. Also, in some rodent models, anxiety- and depression-like behaviors caused by stressors were antagonized by NC [25, 26]. With respect to these paradoxical interactions between NC and stressors, complicated mechanisms underlying the effects of NC, which are associated with a characteristically altered combination of nAChR activation plus desensitization and subsequent modulation of the stress-related neurotransmitter/neuroendocrine systems [3, 4], seemed to be involved, but the details of the relevant mechanisms have not been elucidated. Nevertheless, the data from behavioral studies on the interactions between the stress-related effects of NC and other stressors seem to contribute, at least in part, to understanding the involved mechanisms, predicting the risk of exacerbated NC effects in stressor-exposed smokers, and improving the ability to treat the NC addiction.

“Epigenetics” was originally defined in 1942 as studies on the developmental processes between genotypes and phenotypes [27], and is currently regarded as studies on the reversible regulation of gene expression that occurs throughout the lifecycle of an organism independently of the DNA sequence [28–30]. Epigenetic mechanisms include processes such as DNA methylation, histone modifications (acetylation, methylation, phosphorylation etc.), and alterations in microRNAs (small, non-coding RNAs) [29–32]. Although the epigenetic involvement in the addiction-related effects of NC has not been sufficiently explored, an increasing number of studies suggest a pivotal contribution of epigenetic modifications such as histone acetylation in the brain to the behavioral alterations induced by NC (i.e. conditioned place preference and self-administration) [33, 34]. Furthermore, as recently reviewed, growing evidence suggests that stress-related anxiety and depression are robustly associated with altered epigenetic processes [35, 36].

Among the neurotransmitter systems involved in the effects of NC, in addition to the nicotinic cholinergic and dopaminergic (DAergic) systems that function as fundamental targets [37, 38], an increasing number of studies suggest important roles of the endocannabinoid (ECB) system, which includes cannabinoid (CB) receptors such CB1 and CB2 receptors and the endogenous ligands for these receptors, in NC addiction [39, 40]. Molecularly, overlapping distributions of CB1 and nACh receptors in some brain regions, and functional interactions between these receptors have been reported [41, 42]. Coexpression of CB1 and dopamine (DA) receptors in distinct brain regions, as well as their functional interactions, has also been reported [43, 44]. Furthermore, modulation of the ECB system controls the nAChR-mediated DA release evoked by NC [45]. In rodents, NC-induced anxiety- and depression-related behaviors were modulated by CB ligands, although the effects were different depending on the condition of NC administration [46–50]. The crucial involvement of the ECB system has also been reported in stress-related emotional symptoms including anxiety and depression [51–53]. Epigenetically, the involvement of decreased histone acetylation has been reported in the repressed transcription of the striatal CB1 receptor gene in a mouse model of Huntington’s disease [54]. On the other hand, in the hippocampus and neocortex of neonatal mice, the involvement of ethanol-induced amplification of histone acetylation in the exon region of the CB1 receptor gene, which enhanced the function of CB1 receptors, has been reported in the memory-related neurobehavioral abnormalities after growth [55]. In the prefrontal cortex of adolescent rats, increased histone acetylation was induced by the CB1 agonist Δ(9)-tetrahydrocannabinol (THC) [56]. To date, however, epigenetic processes directly associated with the involvement of the ECB system in the effects of NC and/or stressors have not been demonstrated. Some experimental studies showed antagonistic effects of CB agonists against both histone modifications (phosphorylation or phosphoacetylation) and behavioral abnormalities (seizures or dyskinesias) mediated by the neurotransmitter systems related to NC and/or stressors (e.g. DAergic system) [57, 58]. Nevertheless, the role of histone acetylation, a representative epigenetic process implicated in the behavioral effects of NC and stressors [33–36], in the interacting effects of NC and/or stressors with the ECB system has not been investigated.

In the present study, using behavioral tests in mice (elevated plus-maze (EPM) and forced swimming (FS) test), anxiety- and depression-related behavioral alterations caused by NC and/or immobilization stress (IM), a typical stressor, were investigated, considering the epigenetic involvement of histone acetylation as previously reported [33–36, 59, 60]. Moreover, based on the above-suggested contributing roles of the ECB system and possibility of epigenetic involvement [54–58], the interacting influence of selected CB1 ligands on the effects of histone deacetylase (HDAC) inhibitors that mainly induce histone acetylation, possibly on the therapeutic effects, was evaluated.

## Materials and Methods

### Subjects and Ethics Statement

Based on previous studies on NC and stressor treatments [47, 50], male ICR mice (80 ± 10 days old) (Shizuoka Laboratory Animal Center, Hamamatsu, Japan) were housed in a forced-air facility, which was maintained at 23°C and 50% relative humidity, with a 12 h/12 h light/dark cycle. The mice were kept separately in single transparent cages measuring 23.5 × 16.5 × 12 cm, and were allowed water and rodent chow *ad libitum*. The experiments described in this report were approved by the Animal Care and Use Committees of Kyoto University, and were conducted in accordance with the “Regulation on Animal Experimentation at Kyoto University” of the institution (established in 2007 and updated in 2013) [61], which is based on the National Institutes of Health Guide for the Care and Use of Laboratory Animals. All efforts were made by trained personnel in order to minimize the pain experienced by the mice. No mice died during the experiments. All of the observations and evaluations were performed by a trained observer who was blinded to and not informed of the treatment conditions in advance. Each experimental group contained 10 mice.

### Drug and Stressor Treatments

The protocols for the NC and stressor treatments were determined based on preliminary experiments and previous studies [47, 50, 62]. With respect to NC, repeated subcutaneous (s.c.) doses of NC that caused the emotional behaviors (anxiety- and depression-like behaviors) most effectively in mice [50] were selected: a single s.c. dose of 0.8 mg/kg was administered daily for 4 days. NC (Nacalai Tesque, Inc., Kyoto, Japan) was supplied in free-base form at 95% purity, and was freshly dissolved in saline to a volume of 5 ml/kg immediately before each administration. With respect to the stressor, treatments using IM, which have also been demonstrated to cause these emotional behaviors in rodents [50, 63], were used. In the present experiments, repeated IM treatments in which the effects were almost equivalent to the peak effects of the NC treatments in preliminary experiments were selected: 10 min of IM, which was induced by placing the mouse in a narrow space (diameter about 12 cm) in a vinyl bag with some breathing holes, was performed once per day for 4 days. Furthermore, to investigate the interactions between NC and IM, the behavioral alterations were examined in the NC plus IM group (NC-IM group) which received the above s.c. dose of NC 10 min before the IM treatment once per day for 4 days, according to a previously reported study [64].

The HDAC inhibitors sodium butyrate (SB) and valproic acid (VA), the selective CB1 agonist ACPA (arachidonylcyclopropylamide; AC), and the selective CB1 antagonist SR 141716A (N-(Piperidin-1-yl)-5-(4-chlorophenyl)-1-(2,4-dichlorophenyl)-4-methyl-1H-pyrazole-3-carboxamide hydrochloride; SR) were purchased from Tocris Cookson Inc. (Ellisville, Missouri, USA), and the doses were selected based on previous studies and preliminary experiments [65–70]. For each drug, the data were collected and shown for those intraperitoneal (i.p.) doses that induced no toxic behavioral alterations by themselves at the prescribed time point: 50, 100 and 200 mg/kg for SB, 200, 300 and 400 mg/kg for VA, 0.05, 0.2 and 1 mg/kg for AC, and 0.5, 1 and 2 mg/kg for SR. Furthermore, in the experiments examining the interacting role of HDAC inhibition (histone acetylation) with the ECB system, the CB1 antagonist SR was used in combination with the effective HDAC inhibitors, based on previous studies [66,68]. The drugs were dissolved and diluted using a mixed solution of dimethylsulphoxide (DMSO) plus distilled water, and were administered in a volume of 2.5 ml/kg 60 min (SB, AC and SR) or 30 min (VA) before each NC, IM or NC-IM treatment, based on previous data and preliminary experiments [65–70]. In the HDAC inhibitor- or CB1 ligand-only groups, equivolume saline vehicle was injected instead of the NC, IM or NC-IM treatment. In the control group without any drug or stressor treatment (control group), the mixed vehicle solution of DMSO and distilled water was injected instead of the CB1 ligands, and then equivolume saline vehicle was injected instead of the NC, IM or NC-IM treatment. The drug and stressor treatments and each experimental session were performed between 12 h and 16 h of the light cycle.

### Behavioral Tests

#### Elevated plus-maze (EPM) test

Based on previous studies [46, 50, 71–73], alterations in anxiety-related behaviors were examined in the EPM test, using a cardboard apparatus that consisted of two opposite open arms 50 × 10 cm (length and width) and two enclosed arms 50 × 10 × 30 cm (length, width, and height), positioned 50 cm from the floor. After the number of entries into open arms, the time spent on open arms (sec), and the total number of entries into arms were evaluated (5 min test periods), the percentage of entries into open arms and the percentage of time spent on open arms were calculated as parameters of anxiety-related behaviors. The total number of entries into arms was assessed as a parameter representing locomotor activity [72]. Based on previous data [50], the evaluations of these parameters were performed at the 2 h time point after the last NC, IM or NC-IM treatment. At the beginning of each experimental session, each mouse was placed diagonally in the center platform of the maze, facing both the open and enclosed arms [50].

#### Forced swimming (FS) test

Based on previous studies [63, 74, 75], alterations in depression-related behaviors were examined in the FS test, using a glass cylinder apparatus 33 cm in height and 18 cm in diameter containing 14 cm of water at 21–23°C and the activity-measuring and recording system Supermex-CompACT AMS instrument (Muromachi Kikai Co. Ltd., Tokyo, Japan), for which an infrared sensor was placed over the cylinder at a distance of 20 cm from the water and the frequency of each mouse crossing the area under the sensor while swimming was measured as a number of counts. As parameters of the test, the time until immobility, that is, the time after when only modest swimming behaviors necessary to avoid drowning (<60 counts/min under the present conditions), and the activity counts (per 10 min) which reflected the amount of swimming behaviors during a 10 min experimental period were monitored. Considering the time course of the behavioral alterations [47], the evaluations of these parameters were performed at the 2 h time point after the last NC, IM or NC-IM treatment.

### Statistical analysis

The data were subjected to two- or three-way analysis of variance (ANOVA) for each experiment. With respect to the experiments examining the NC- and/or IM-induced anxiety- and depression-related behavioral alterations and the effects of HDAC inhibitors or CB1 ligands, a 2 (NC versus vehicle) × 2 (IM versus vehicle) or 2 (NC versus vehicle) × 2 (IM versus vehicle) × 4 (three doses of each HDAC inhibitor or CB1 ligand versus vehicle) factorial design was used [76, 77]. With respect to the experiments examining the interacting role of HDAC inhibition (histone acetylation) with the ECB system, a 4 (NC, IM, NC-IM versus vehicle) × 2 (most effective dose of each HDAC inhibitor or CB1 ligand versus vehicle) × 4 (three doses of the CB1 antagonist SR versus vehicle) factorial design was used [76, 77]. For pairwise comparisons, Bonferroni post-hoc tests were performed [76]. All of the comparisons were performed using statistical software packages (“Excel Statistics” from Social Survey Research Information Co. Ltd., Tokyo, Japan). P values less than 0.05 were considered to be statistically significant.

## Results

### Antagonistic effects of HDAC inhibitors and CB1 agonist against NC- and/or IM-induced anxiety-like behavioral alterations in the elevated plus-maze (EPM) test

**Fig 1:**
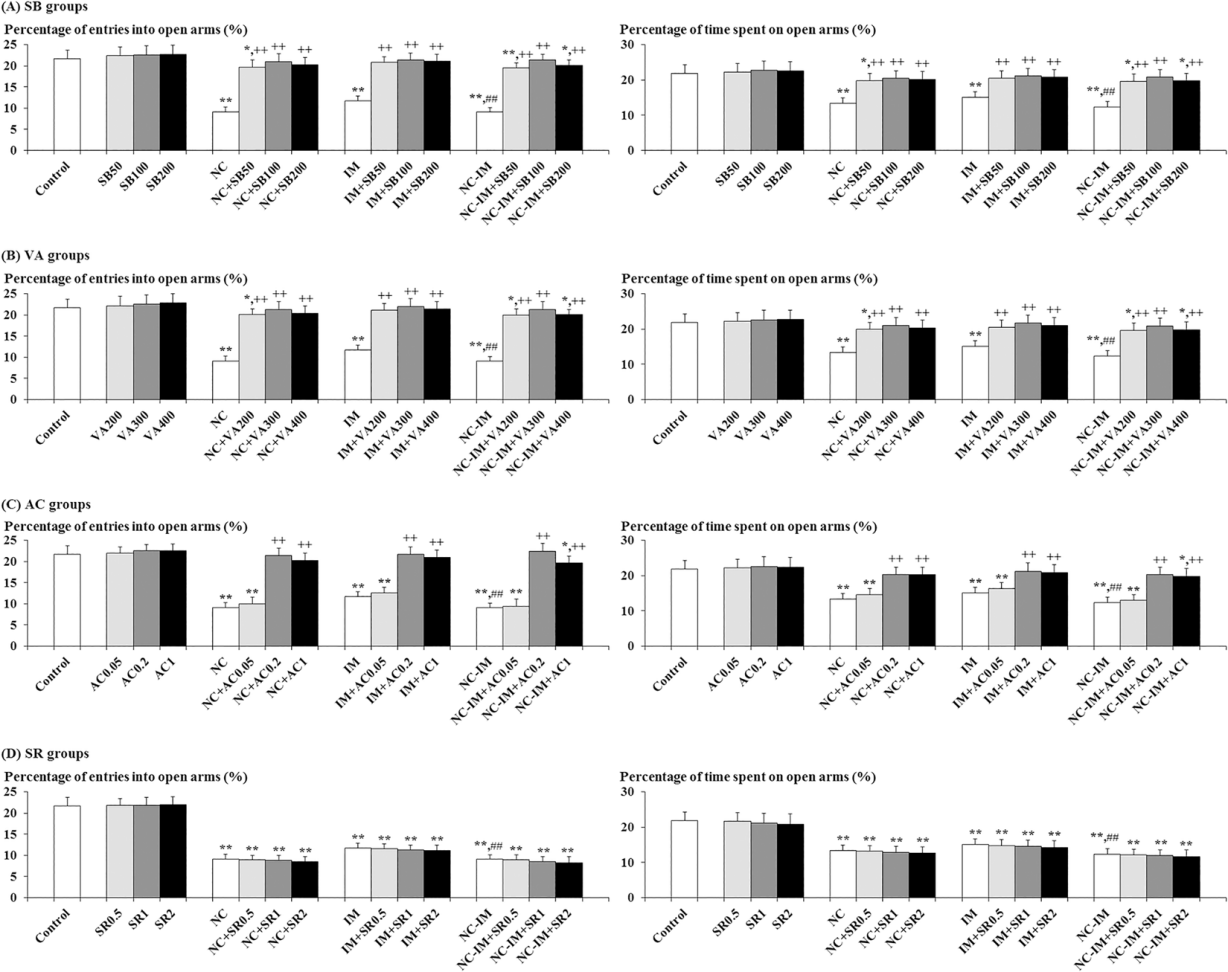
Antagonistic effects of histone deacetylase (HDAC) inhibitors or cannabinoid type 1 (CB1) agonist against anxiety-like behaviors. The parameter values of the elevated plus-maze test at the 2 h time point after the last nicotine (NC) (0.8 mg/kg, s.c.) or immobilization stress (IM) (10 min) treatment are shown as means ± S.D. (n = 10) for each HDAC inhibitor or CB1 ligand co-treatment group (with each i.p. dose (mg/kg)). (A) Sodium butyrate (SB) co-treatment groups (SB groups); (B) Valproic acid (VA) co-treatment groups (VA groups); (C) ACPA (arachidonylcyclopropylamide; AC) co-treatment groups (AC groups); (D) SR 141716A (N-(Piperidin-1-yl)-5-(4-chlorophenyl)-1-(2,4-dichlorophenyl)-4-methyl-1H-pyrazole-3-carboxamide hydrochloride; SR) co-treatment groups (SR groups). The data for the control, NC, IM, and NC plus IM (NC-IM) groups without any HDAC inhibitor or CB1 ligand co-treatments, as well as the HDAC inhibitor- and CB1 ligand-only groups, are also shown. * (p<0.05), ** (p<0.01): significant attenuation as compared to the control group; ++ (p<0.01): significant increase as compared to the NC, IM, or NC-IM group without any co-treatments; ## (p<0.01): significant attenuation as compared to the IM group without any co-treatments.

In both NC, IM and NC-IM groups, at the 2 h time point after the last treatment, anxiety-like behavioral alterations, i.e. statistically significantly attenuated percentage of entries into open arms and significantly attenuated percentage of time spent on open arms, were observed in the EPM test (Fig 1). This is consistent with the results of the ANOVA revealing statistically significant main effects of NC (F(1, 36) = 302.48, P<0.001 for the percentage of entries into open arms and F(1, 36) = 102.70, P<0.001 for the percentage of time spent on open arms) and IM (F(1, 36) = 131.82, P<0.001 for the percentage of entries into open arms and F(1, 36) = 50.58, P<0.001 for the percentage of time spent on open arms). For the NC-IM group, the parameter values were significantly attenuated as compared to the IM group, which is consistent with the results of the ANOVA revealing significant interactions between the NC and IM treatment for each parameter value (F(1, 36) = 133.23, P<0.001 for the percentage of entries into open arms and F(1, 36) = 25.38, P<0.001 for the percentage of time spent on open arms).

Against these anxiety-like behavioral alterations, statistically significant antagonistic effects, i.e. recoveries from both attenuated percentage of entries into open arms and attenuated percentage of time spent on open arms, were observed in both NC, IM and NC-IM groups co-treated with the HDAC inhibitor SB (50–200 mg/kg), VA (200–400 mg/kg) or the CB1 agonist AC (0.2–1 mg/kg) (Fig 1A–1C). This is consistent with the results of the ANOVA revealing statistically significant main effects of SB (F(3, 144) = 258.06, P<0.001 for the percentage of entries into open arms and F(3, 144) = 59.25, P<0.001 for the percentage of time spent on open arms), VA (F(3, 144) = 237.24, P<0.001 for the percentage of entries into open arms and F(3, 144) = 62.75, P<0.001 for the percentage of time spent on open arms), and AC (F(3, 144) = 374.26, P<0.001 for the percentage of entries into open arms and F(3, 144) = 71.28, P<0.001 for the percentage of time spent on open arms). Furthermore, significant interactions between all of the following treatments were observed: SB versus NC (F(3, 144) = 35.67, P<0.001 for the percentage of entries into open arms and F(3, 144) = 9.57, P<0.001 for the percentage of time spent on open arms), SB versus IM (F(3, 144) = 17.55, P<0.001 for the percentage of entries into open arms and F(3, 144) = 5.26, P<0.01 for the percentage of time spent on open arms), VA versus NC (F(3, 144) = 31.57, P<0.001 for the percentage of entries into open arms and F(3, 144) = 9.36, P<0.001 for the percentage of time spent on open arms), VA versus IM (F(3, 144) = 16.67, P<0.001 for the percentage of entries into open arms and F(3, 144) = 5.42, P<0.01 for the percentage of time spent on open arms), AC versus NC (F(3, 144) = 61.46, P<0.001 for the percentage of entries into open arms and F(3, 144) = 11.80, P<0.001 for the percentage of time spent on open arms), and AC versus IM (F(3, 144) = 28.57, P<0.001 for the percentage of entries into open arms and F(3, 144) = 6.51, P<0.001 for the percentage of time spent on open arms). In each group co-treated with the CB1 antagonist SR, as well as in each HDAC inhibitor- or CB1 ligand-only group, no significant alterations as compared to the control group were observed for each parameter value under the present experimental conditions.

### Antagonistic effects of HDAC inhibitors and CB1 agonist against NC- and/or IM-induced depression-like behavioral alterations in the forced swimming (FS) test

**Fig 2:**
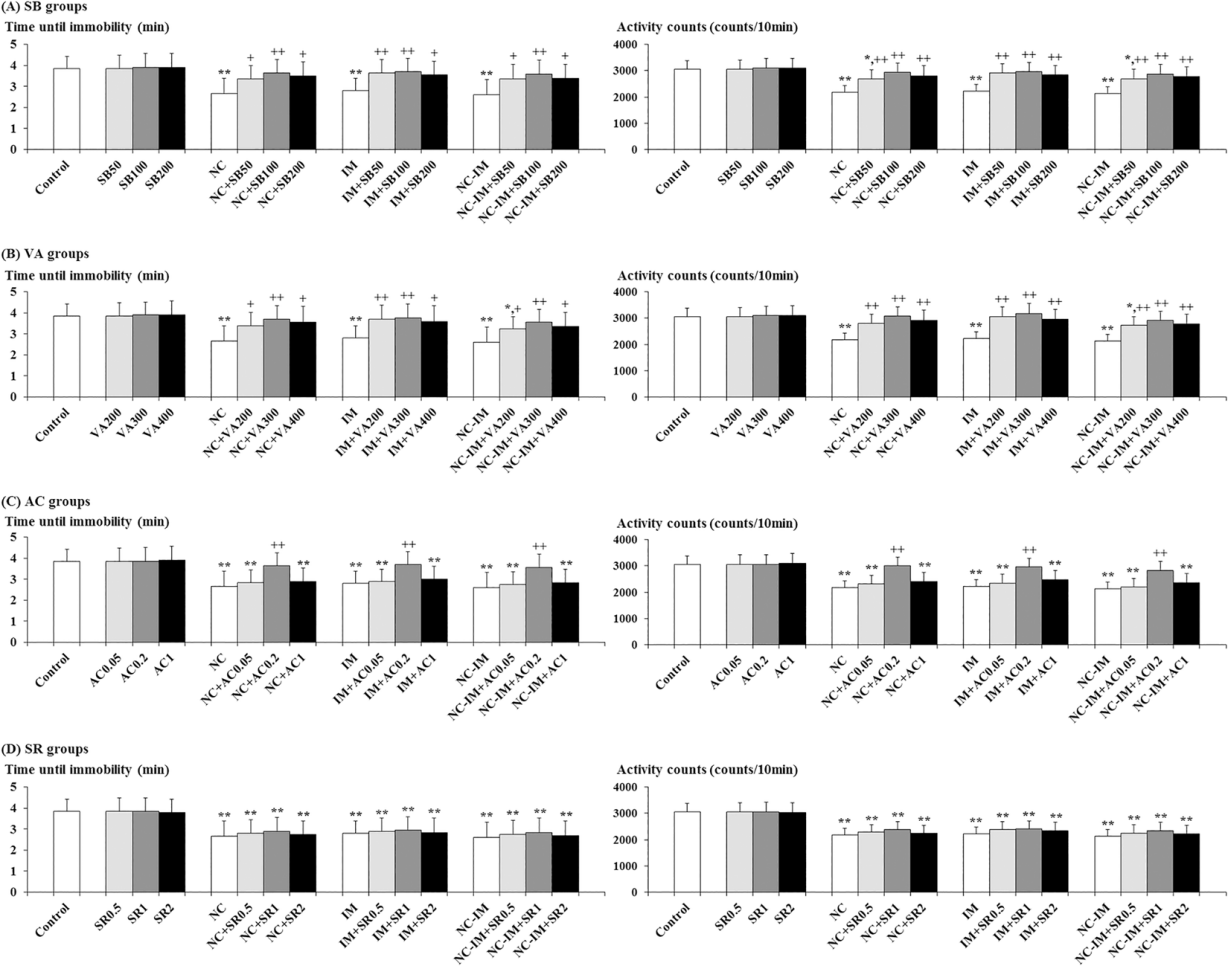
Antagonistic effects of histone deacetylase (HDAC) inhibitors or cannabinoid type 1 (CB1) agonist against depression-like behaviors. The parameter values of the forced swimming test at the 2 h time point after the last nicotine (NC) (0.8 mg/kg, s.c.) or immobilization stress (IM) (10 min) treatment are shown as means ± S.D. (n = 10) for each HDAC inhibitor or CB1 ligand co-treatment group (with each i.p. dose (mg/kg)). (A) Sodium butyrate (SB) co-treatment groups (SB groups); (B) Valproic acid (VA) co-treatment groups (VA groups); (C) ACPA (arachidonylcyclopropylamide; AC) co-treatment groups (AC groups); (D) SR 141716A (N-(Piperidin-1-yl)-5-(4-chlorophenyl)-1-(2,4-dichlorophenyl)-4-methyl-1H-pyrazole-3-carboxamide hydrochloride; SR) co-treatment groups (SR groups). The data for the control, NC, IM, and NC plus IM (NC-IM) groups without any HDAC inhibitor or CB1 ligand co-treatments, as well as the HDAC inhibitor- and CB1 ligand-only groups, are also shown. * (p<0.05), ** (p<0.01): significant attenuation as compared to the control group; + (p<0.05), ++ (p<0.01): significant increase as compared to the NC, IM, or NC-IM group without any co-treatments.

In both NC, IM and NC-IM groups, at the 2 h time point after the last treatment, depression-like behavioral alterations, i.e. statistically significantly attenuated time until immobility and significantly attenuated activity counts which reflected both the overall activity during the swim behaviors and the minimum activity after immobility, were observed in the FS test (Fig 2). This is consistent with the results of the ANOVA revealing statistically significant main effects of NC (F(1, 36) = 9.77, P<0.01 for the time until immobility and F(1, 36) = 29.84, P<0.001 for the activity counts) and IM (F(1, 36) = 6.03, P<0.05 for the time until immobility and F(1, 36) = 22.69, P<0.001 for the activity counts). For the NC-IM group, the parameter values were not significantly different from either NC- or IM-only group (Fig 2).

Against these depression-like behavioral alterations, statistically significant antagonistic effects, i.e. recoveries from both attenuated time until immobility and attenuated activity counts, were observed in both NC, IM and NC-IM groups co-treated with SB (50–200 mg/kg), VA (200–400 mg/kg) or AC (0.2 mg/kg) (Fig 2A–2C). This is consistent with the results of the ANOVA revealing statistically significant main effects of SB (F(3, 144) = 8.92, P<0.001 for the time until immobility and F(3, 144) = 20.03, P<0.001 for the activity counts), VA (F(3, 144) = 9.12, P<0.001 for the time until immobility and F(3, 144) = 26.35, P<0.001 for the activity counts), and AC (F(3, 144) = 8.86, P<0.001 for the time until immobility and F(3, 144) = 20.83, P<0.001 for the activity counts). In each group co-treated with the CB1 antagonist SR, as well as in each HDAC inhibitor- or CB1 ligand-only group, no significant alterations as compared to the control group were observed for each parameter value under the present experimental conditions.

### Interacting effects between HDAC inhibitors and CB1 antagonist

In order to investigate the interacting role of HDAC inhibitors with the ECB system, interactions with the CB1 antagonist SR (0.5, 1 and 2 mg/kg) were examined for the most effective dose of the HDAC inhibitors SB (100 mg/kg) and VA (300 mg/kg). For comparison, interactions with the same doses of SR were examined for the most effective dose of the CB1 agonist AC (0.2 mg/kg).

**Fig 3:**
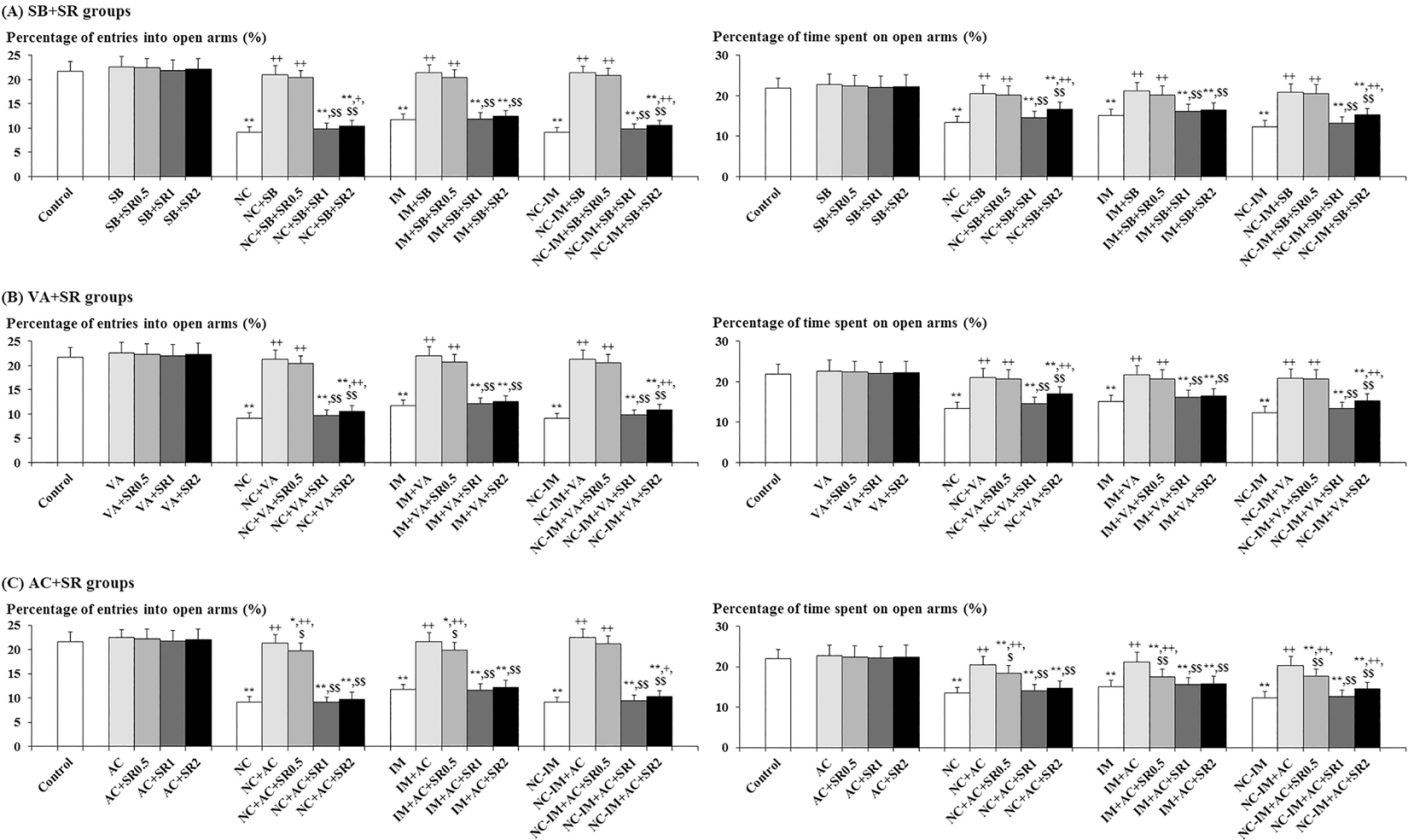
Interacting effects between cannabinoid type 1 (CB1) antagonist (SR 141716A) and efficacious (anxiolytic-like) histone deacetylase (HDAC) inhibitors or CB1 agonist against anxiety-like behavioral alterations caused by nicotine (NC) and/or immobilization stress (IM). The parameter values of the elevated plus-maze test at the 2 h time point after the last NC (0.8 mg/kg, s.c.) or IM (10 min) treatment are shown as means ± S.D. (n = 10) for each HDAC inhibitor or CB1 ligand “plus” SR 141716A (N-(Piperidin-1-yl)-5-(4-chlorophenyl)-1-(2,4-dichlorophenyl)-4-methyl-1H-pyrazole-3-carboxamide hydrochloride; SR) co-treatment group (with each i.p. dose (mg/kg)). (A) Sodium butyrate (SB) plus SR co-treatment groups (SB+SR groups); (B) Valproic acid (VA) plus SR co-treatment groups (VA+SR groups); (C) ACPA (arachidonylcyclopropylamide; AC) plus SR co-treatment groups (AC+SR groups). The data for the control, NC, IM, and NC plus IM (NC-IM) groups without any HDAC inhibitor or CB1 ligand co-treatments, as well as the HDAC inhibitor-, CB1 agonist-, HDAC inhibitor plus SR-, and CB1 agonist plus SR-only groups, are also shown. * (p<0.05), ** (p<0.01): significant attenuation as compared to the control group; + (p<0.05), ++ (p<0.01): significant increase as compared to the NC, IM, or NC-IM group without any co-treatments; $ (p < 0.05), $ $ (p < 0.01): significant attenuation as compared to the NC, IM, or NC-IM group co-treated with the efficacious HDAC inhibitor or CB1 agonist.

Against the anxiolytic-like effects of SB and VA in the EPM test, as well as against those effects of AC, significant antagonistic effects were provided by SR (1 and 2 mg/kg) for each parameter in both NC, IM and NC-IM groups (Fig 3). These data are consistent with the results of ANOVA revealing statistically significant interactions of the following treatments: NC and/or IM × SB × SR (F(9, 288) = 16.53, P<0.001 for the percentage of entries into open arms and F(9, 288) = 3.10, P<0.01 for the percentage of time spent on open arms), NC and/or IM × VA × SR (F(9, 288) = 15.16, P<0.001 for the percentage of entries into open arms and F(9, 288) = 3.25, P<0.001 for the percentage of time spent on open arms), and NC and/or IM × AC × SR (F(9, 288) = 17.69, P<0.001 for the percentage of entries into open arms and F(9, 288) = 2.93, P<0.01 for the percentage of time spent on open arms).

**Fig 4:**
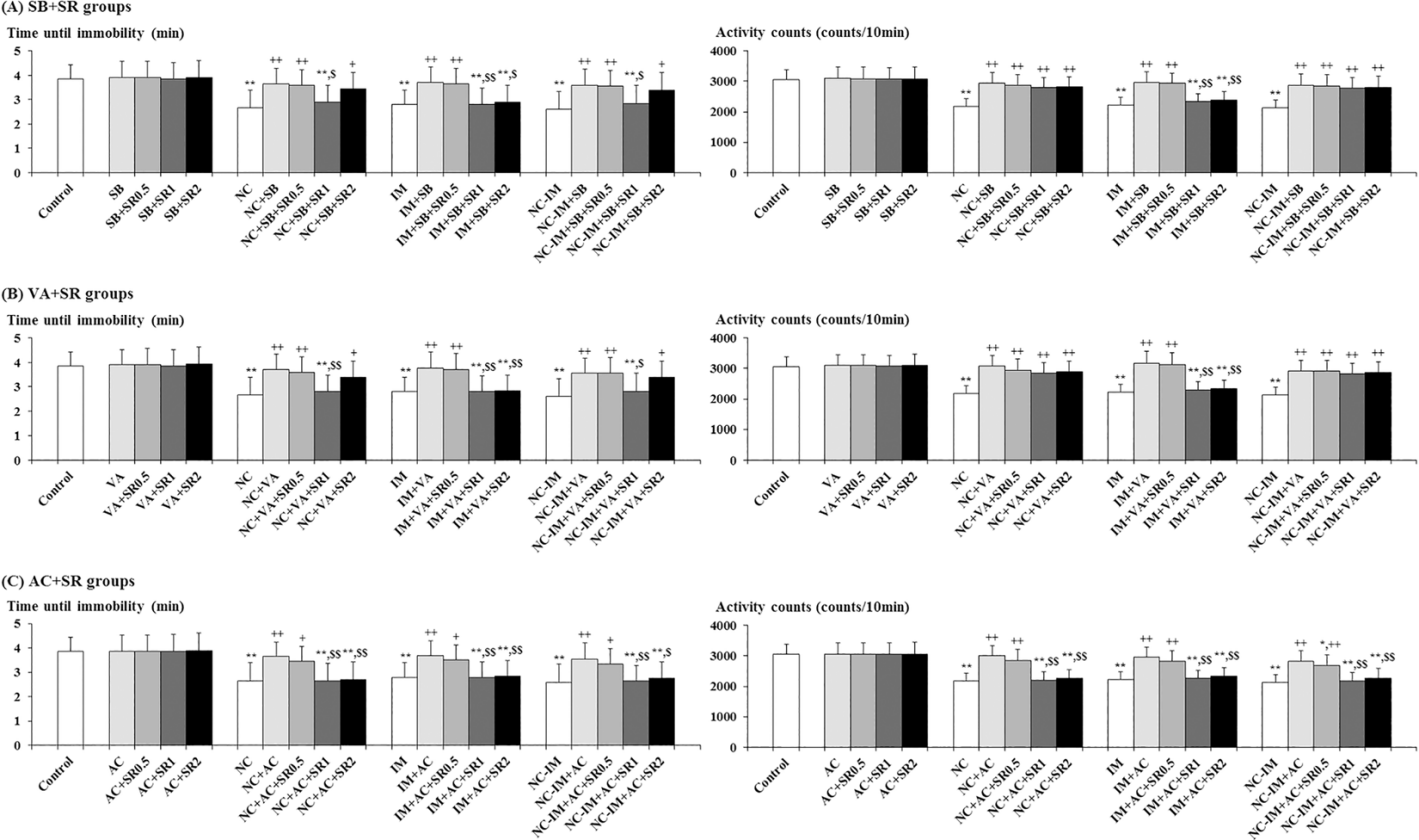
Interacting effects between cannabinoid type 1 (CB1) antagonist (SR 141716A) and efficacious (antidepressant-like) histone deacetylase (HDAC) inhibitors or CB1 agonist against depression-like behavioral alterations caused by nicotine (NC) and/or immobilization stress (IM). The parameter values of the forced swimming test at the 2 h time point after the last NC (0.8 mg/kg, s.c.) or IM (10 min) treatment are shown as means ± S.D. (n = 10) for each HDAC inhibitor or CB1 ligand “plus” SR 141716A (N-(Piperidin-1-yl)-5-(4-chlorophenyl)-1-(2,4-dichlorophenyl)-4-methyl-1H-pyrazole-3-carboxamide hydrochloride; SR) co-treatment group (with each i.p. dose (mg/kg)). (A) Sodium butyrate (SB) plus SR co-treatment groups (SB+SR groups); (B) Valproic acid (VA) plus SR co-treatment groups (VA+SR groups); (C) ACPA (arachidonylcyclopropylamide; AC) plus SR co-treatment groups (AC+SR groups). The data for the control, NC, IM, and NC plus IM (NC-IM) groups without any HDAC inhibitor or CB1 ligand co-treatments, as well as the HDAC inhibitor-, CB1 agonist-, HDAC inhibitor plus SR-, and CB1 agonist plus SR-only groups, are also shown. * (p<0.05), ** (p<0.01): significant attenuation as compared to the control group; + (p<0.05), ++ (p<0.01): significant increase as compared to the NC, IM, or NC-IM group without any co-treatments; $ (p < 0.05), $ $ (p < 0.01): significant attenuation as compared to the NC, IM, or NC-IM group co-treated with the efficacious HDAC inhibitor or CB1 agonist.

Against the antidepressant-like effects of SB and VA in the FS test, significant antagonistic effects were provided by SR (1 and 2 mg/kg) for each parameter in the IM groups (Fig 4A and 4B), which is consistent with the results of the ANOVA revealing statistically significant interactions of treatments for both SB versus SR (F(3, 288) = 4.67, P<0.01 for the time until immobility and F(3, 288) = 4.28, P<0.01 for the activity counts) and VA versus SR (F(3, 288) = 5.42, P<0.01 for the time until immobility and F(3, 288) = 6.75, P<0.001 for the activity counts). However, in the NC and NC-IM groups, only limited antagonistic effects (i.e. significant antagonistic effects only against recovered time until immobility) were provided by 1 mg/kg SR against the effects of SB and VA (Fig 4A and 4B). On the other hand, against the antidepressant-like effects of AC, significant antagonistic effects were provided by SR (1 and 2 mg/kg) for each parameter in both NC, IM and NC-IM groups (Fig 4C), which is consistent with the results of the ANOVA revealing statistically significant interactions of treatments for AC versus SR (F(3, 288) = 6.52, P<0.001 for the time until immobility and F(3, 288) = 17.37, P<0.001 for the activity counts).

## Discussion

### NC- and/or IM-induced anxiety- and depression-like behavioral alterations and antagonistic effects of HDAC inhibitors

In the NC group receiving repeated treatments of 0.8 mg/kg NC, as well as in the IM group, anxiety- and depression-like behavioral alterations were observed in the EPM and FS tests under the present experimental conditions (Figs 1 and 2), which supports the data in previous studies [47, 50, 63]. Although the opposite effects on anxiety and depression have been reported for NC depending on the experimental condition [3, 13–17, 46–50, 78], anxiogenic- and depressogenic-like effects like those observed with the IM treatment were observed with the NC treatment in the present study. In the author’s preliminary experiments, consistent with several previous studies [25, 47, 50, 63, 75, 78, 79], even acute 1-day treatment of NC or IM induced both anxiety- and depression-like behaviors in mice. However, with the repeated 4-day treatment used in the present study, enhanced anxiety- and depression-like behaviors as compared to the acute 1-day treatment were observed at the selected time point (2 h) after the last treatment. Yet, this mouse model of subacute treatment with a small number of repeated doses did not seem to sufficiently reflect the human cases of daily and/or dependent smoking who are suffering from anxiety and depression [5–10]. Nevertheless, since recent studies have reported that some brain dysfunction is caused by small amounts of NC intake even in non-smoking humans [80–82], it is possible that the present results on NC-induced anxiety and depression in mice mimic some kind of latent negative influence on emotion-related brain function in humans.

As reviewed previously, the involvement of stress-related neurotransmitter systems, such as the DAergic and serotonergic systems (DA and serotonin receptors), has been reported in the development of anxiety and depression: neuroimaging and pharmacological studies have demonstrated that the dysfunction of DA and serotonin receptors is associated with increased anxiety and depression [83–86]. Furthermore, the combined influence of the nicotinic cholinergic system (nAChRs) and DAergic system, both of which also function as targets for NC, was closely correlated with the elicitation of anxiety- and depression-like behavioral responses [87–89]. The nicotinic cholinergic system also cooperated with the serotonergic system in modulating anxiety and depression [88, 90, 91]. In addition to the neurotransmitter systems, several stress-related neuromolecular responses, such as those of the neuroendocrine system (e.g. secretion of hypothalamic-pituitary-adrenal (HPA)-axis hormones such as corticosterone, norepinephrine, etc.), immediate early gene (IEG) (e.g. Arc, c-Fos, etc.) expression and dysregulated hippocampal neurogenesis, have been reported to accompany and participate in the control of anxiety- and depression-like behaviors, in which the involvement of the DAergic and serotonergic systems, as well as the nicotinic cholinergic system, has been suggested [92–104]. Similar modifications in these neuromolecular responses were also induced by NC in some rodent experimental models [105–107]. Moreover, as discussed later, these neuromolecular responses and the modulation of the stress-related neurotransmitter systems were associated with epigenetic histone acetylation.

With respect to interactions between NC and IM in the NC-IM group, statistically significant enhancement of anxiogenic-like effects was caused by NC plus IM as compared to the IM-only group in the EPM test (Fig 1). In the FS test, no significant alterations in depressogenic-like effects were observed (Fig 2). While the relationship between stressors such as IM and NC in the behavioral effects is controversial and “antistress” effects of pre-exposed NC (cigarette) have also been reported depending on the condition [24–26], significant synergistic effects like those observed in previous studies [21–23] were provided by the IM plus NC treatment in the present anxiety-related experimental model. An augmented increase in secreted HPA-axis hormones and immediate early gene expression, which accompanied the enhanced behavioral effects, has also been reported in several previous studies [108, 109]. Nevertheless, in the depression-related behavioral test (FS test), no significant interacting effects between IM and NC were observed for the behavioral alterations. Although the molecular mechanisms underlying these differences in NC/IM interactions between anxiety- and depression-related behavioral alterations were not clarified, decreased interacting effects of stressors against NC-induced behaviors have been reported depending on the type of NC and/or stressor treatment, assessed type of behavioral response and genotype [110–112]. The blunted interacting effects for neuromolecular responses were also suggested depending on the experimental condition [110–112]. Moreover, considering the additional interactions with the HDAC inhibitors (Figs 1 and 2) and putative influence of histone acetylation in the NC-IM group, there is a possibility that the incongruous interactions between NC and IM in the present study were closely associated with multiple molecular modifications at the epigenetic level.

The NC- and/or IM-induced anxiety- and depression-like behavioral alterations were antagonized by the HDAC inhibitors SB and VA (Figs 1 and 2). In previous studies, anxiogenic- and depressogenic-like effects of various stressors were antagonized by SB and VA [60, 67, 113, 114]. From the present results, the epigenetic involvement of histone acetylation in the elicitation of NC-induced anxiety- and depression-like behaviors was also suggested. Molecularly, decreased histone acetylation induced in some brain regions (e.g. hippocampus and nucleus accumbens) by stressors was involved in the dysregulation of the stress-related neurotransmitter systems such as the DAergic and serotonergic systems (e.g. decreased synthesis of DA and serotonin), as well as anxiety- and depression-like behavioral alterations, and HDAC inhibitors regulatorily antagonized both molecular and behavioral effects [115, 116]. Although the contribution of histone acetylation to the modulated function of the direct NC target nAChRs has not been fully elucidated, reduced histone acetylation at the promoters of the gene encoding the acetylcholine-hydrolyzing enzyme acetylcholinesterase (AChE), which seemed to dysregulate the function of the nicotinic cholinergic system, has been reported in the hippocampus of stressor-treated mice eliciting anxiety-like behaviors, and HDAC inhibition mediated by gene manipulation regulatorily abolished both AChE-related effects and stress-related anxiety [117]. Moreover, in rats treated with repeated NC, increased histone acetylation was observed at several promoters of the gene encoding DA receptors, which function as NC target receptors, resulting in increased expression of the DA receptor gene in the prefrontal cortex, one of the target areas for anxiolytics and antidepressants that is also closely associated with the nicotinic cholinergic system [118]. Furthermore, the involvement of histone acetylation in the above-mentioned stress-related responses such as modulated secretion of HPA-axis hormones, IEG expression, and hippocampal neurogenesis has been reported, and accompanying modulation of anxiety- and depression-related behavioral symptoms has been suggested [119–125]. Although the influence of histone acetylation varied depending on the type and duration of stressors, decreased acetylation was directly induced by stressors in several experimental models [124, 125], and thus “anti-stress” effects of increased acetylation provided by HDAC inhibitors were predicted. In the present study, the antagonistic effects of the HDAC inhibitors SB and VA against the stressor (including NC)-induced anxiety- and depression-like behaviors were not dose-dependent. The mechanism underlying this dose-response has not been clarified, but seemed to be correlated with the attenuated anti-stress effects reported for high doses of the HDAC inhibitors, and the modulated involvement of related neurotransmitter systems (e.g. DAergic, GABAergic and glutamatergic systems) was suggested [123, 126].

### Effects of ECB ligands against NC- and/or IM-induced anxiety- and depression-like behavioral alterations and putative epigenetic interactions with HDAC inhibitors

In the present experimental model, the selective CB1 agonist AC antagonized the NC- and/or IM-induced anxiety- and depression-like behavioral alterations (Figs 1 and 2). These results were predictable considering the above-mentioned potent controlling roles of the ECB system in the effects of NC and stressors [39, 40, 51–53], and were consistent with the central CB1 agonist-induced anxiolytic- and antidepressant-like effects provided against both NC- and stressor-induced behavioral alterations in a number of previous rodent studies [46, 48, 50, 127–130]. Neuroanatomical overlap and functional interactions between the ECB system (CB1 receptors) and NC- and/or stress-related neurotransmitter systems (e.g. nicotinic cholinergic, DAergic and serotonergic systems) also support the present results [41–45, 52, 53, 131]. Furthermore, the activation of the ECB system was involved in the attenuation of the stress-related neuromolecular responses accompanying anxiety- and depression-like behaviors (e.g. responses of the neuroendocrine system, c-fos expression and decreased hippocampal neurogenesis), which seemed to be associated with epigenetic histone acetylation [51, 132–135]. In the present study, obvious dose-dependent effects were not observed for CB1 agonists, and seemed to be due to the mechanisms related to multiple neurotransmitter systems including both ECB and stress-related neurotransmitter systems involved in anti-anxiety and/or anti-depression (e.g. GABAergic, glutamatergic and serotonergic systems) [129, 130, 136, 137]. In previous studies, depending on the treatment condition, the anxiogenic- and depressogenic-like effects of NC or stressors were enhanced or accompanied by the activation of the ECB system and antagonized by CB antagonists such as SR, in which the varying involvement of the relevant neurotransmitter systems such as GABAergic, glutamatergic and serotonergic systems, in addition to the ECB system, has been suggested [127, 130, 137–143]. However, under the present condition, anxiolytic- and antidepressant-like effects were elicited by the CB1 agonist AC, and these effects were blocked by the co-administration of the CB1 antagonist SR (Figs 3 and 4). Although the non-toxic doses of SR selected in the present study were behaviorally inactive against the NC- and/or IM-induced anxiogenic- and depressogenic-like effects, the blocking effects against AC were provided by each dose. The effects of SR were not dose-dependent and seemed to be correlated with the complicated modulation of the neurotransmitter systems other than the ECB system (e.g. GABAergic and catecholaminergic systems), as previously reported [130, 144, 145].

Like the effects induced by AC, the anxiolytic- and antidepressant-like effects elicited by the HDAC inhibitors SB and VA were to some extent blocked by the co-administration of the CB1 antagonist SR (Figs 3 and 4). From these results, it could be predicted that epigenetic histone acetylation has an important role in both activation of the ECB system and anxiolytic/antidepressant-like behavioral responses. To date, several studies mentioned above investigated the role of histone acetylation in the modulated function of the ECB system (CB1 receptors): increased function of the ECB system was accompanied by increased histone acetylation in the normal brain, but the histone acetylation-induced increase in the expression of CB1 receptors occurred in some pathological (stress-loaded) conditions such as neonatal ethanol treatment [54–56]. Moreover, there have been reports on the contribution of excess histone acetylation-related enhanced function of the ECB system during the neonatal period to impaired memory-related behavioral alterations in adulthood [55], but the involvement of such epigenetically modulated function of the ECB system (CB1 receptors) in the anxiolytic- and antidepressant-like effects induced by HDAC inhibitors has not been examined. With respect to other types of histone modifications (phosphorylation or phosphoacetylation) related to HDAC inhibitors, the association with both function of the ECB system modulated by CB agonists and behavioral alterations involving the stress-related neurotransmitter system (i.e. seizures or dyskinesias related to the DAergic system) has been reported, as mentioned above [57, 58]. Furthermore, based on the present results that some blocking influence was provided by the CB1 antagonist SR against the SB- and VA-induced anxiolytic- and antidepressant-like effects, as well as against the CB1 agonist AC-induced attenuating effects on anxiety and depression, the HDAC inhibitor-induced histone acetylation seemed to contribute to the activation of the ECB system (CB1 receptors), at least indirectly through the modulation of some relevant neurotransmitter systems, and then play an important role in the attenuation of NC- and/or stressor-induced anxiety- and depression-like behaviors. Therefore, some molecular regulatory role of HDAC inhibitor-induced histone acetylation could be hypothesized in both function of the ECB system and stress/emotion-related behaviors, although direct evidence has not yet been demonstrated. In the NC treatment groups (NC and NC-IM groups), the antidepressant-like effects of the HDAC inhibitors SB and VA were antagonized to a limited degree by SR, and only the recovered time until immobility was impacted. It is possible that histone acetylation within the NC-related neurotransmitter systems other than the ECB system (e.g. nACh system) contributed more effectively to the antidepressant-like effects of SB and VA than to the anxiolytic-like effects.

In summary, the present results using HDAC inhibitors (SB and VA) show the involvement of epigenetic histone acetylation in the attenuation of NC- and/or IM-induced anxiety- and depression-like behavioral alterations. The selective CB1 agonist AC, like HDAC inhibitors, provided anxiolytic- and antidepressant-like effects against these behavioral alterations, which suggests the involvement of the ECB system, and the selective CB1 antagonist SR antagonized the effects of AC. Some attenuating influence of SR was also observed on the anxiolytic- and antidepressant-like effects of HDAC inhibitors. From the present results, it could be hypothesized that the HDAC inhibitor-induced histone acetylation was, at least to some extent, simultaneously associated with both function of the ECB system (CB1 receptors), one of the targets for HDAC inhibitors, and stressor (NC- and/or IM)-induced emotion-related (anxiety- and depression-like) behaviors. However, against the antidepressant-like effects of HDAC inhibitors, the attenuating influence of SR was limited in the NC treatment groups (NC and NC-IM groups). In the elicitation of HDAC inhibitor-induced antidepressant-like effects in the NC treatment groups, there may be varying involvement of histone acetylation within the ECB vs. other NC-related neurotransmitter systems, and important therapeutic roles of neurotransmitter systems other than the ECB system at the epigenetic level were also suggested.
